# Closed–loop oxygen usage during invasive mechanical ventilation of pediatric patients (CLOUDIMPP): a randomized controlled cross-over study

**DOI:** 10.3389/fmed.2024.1426969

**Published:** 2024-09-10

**Authors:** Gulhan Atakul, Gokhan Ceylan, Ozlem Sandal, Ekin Soydan, Pinar Hepduman, Mustafa Colak, Jan M. Zimmermann, Dominik Novotni, Utku Karaarslan, Sevgi Topal, Hasan Aǧin

**Affiliations:** ^1^Department of Paediatric Intensive Care Unit, Dr Behcet Uz Children's Disease and Surgery Training and Research Hospital, Health Sciences University, Izmir, Türkiye; ^2^Department of Medical Research, Hamilton Medical AG, Chur, Switzerland; ^3^Department of Paediatric Intensive Care Unit, Aydin Obstetrics and Children Hospital, Health Sciences University, Aydin, Türkiye; ^4^Department of Paediatric Intensive Care Unit, Erzurum Territorial Training and Research Hospital, Health Sciences University, Erzurum, Türkiye; ^5^Department of Paediatric Intensive Care Unit, Cam Sakura Training and Research Hospital, Health Sciences University, Istanbul, Türkiye

**Keywords:** hypoxemia, oxygen therapy, invasive mechanical ventilation, automation, closed-loop, oxygen controller, intensive care, pediatrics

## Abstract

**Background:**

The aim of this study is the evaluation of a closed-loop oxygen control system in pediatric patients undergoing invasive mechanical ventilation (IMV).

**Methods:**

Cross-over, multicenter, randomized, single-blind clinical trial. Patients between the ages of 1 month and 18 years who were undergoing IMV therapy for acute hypoxemic respiratory failure (AHRF) were assigned at random to either begin with a 2-hour period of closed-loop oxygen control or manual oxygen titrations. By using closed-loop oxygen control, the patients' SpO_2_ levels were maintained within a predetermined target range by the automated adjustment of the FiO_2_. During the manual oxygen titration phase of the trial, healthcare professionals at the bedside made manual changes to the FiO_2_, while maintaining the same target range for SpO_2_. Following either period, the patient transitioned to the alternative therapy. The outcomes were the percentage of time spent in predefined SpO_2_ ranges ±2% (primary), FiO_2_, total oxygen use, and the number of manual adjustments.

**Findings:**

The median age of included 33 patients was 17 (13–55.5) months. In contrast to manual oxygen titrations, patients spent a greater proportion of time within a predefined optimal SpO_2_ range when the closed-loop oxygen controller was enabled (95.7% [IQR 92.1–100%] vs. 65.6% [IQR 41.6–82.5%]), mean difference 33.4% [95%–CI 24.5–42%]; *P* < 0.001). Median FiO_2_ was lower (32.1% [IQR 23.9–54.1%] vs. 40.6% [IQR 31.1–62.8%]; *P* < 0.001) similar to total oxygen use (19.8 L/h [IQR 4.6–64.8] vs. 39.4 L/h [IQR 16.8–79]; *P* < 0.001); however, median SpO_2_/FiO_2_ was higher (329.4 [IQR 180–411.1] vs. 246.7 [IQR 151.1–320.5]; *P* < 0.001) with closed–loop oxygen control. With closed–loop oxygen control, the median number of manual adjustments reduced (0.0 [IQR 0.0–0.0] vs. 1 [IQR 0.0–2.2]; *P* < 0.001).

**Conclusion:**

Closed-loop oxygen control enhances oxygen therapy in pediatric patients undergoing IMV for AHRF, potentially leading to more efficient utilization of oxygen. This technology also decreases the necessity for manual adjustments, which could reduce the workloads of healthcare providers.

**Clinical Trial Registration:**

This research has been submitted to ClinicalTrials.gov (NCT05714527).

## Introduction

When it comes to the treatment of respiratory failure of any type, oxygen is a fantastic drug to use. The Pediatric Mechanical Ventilation Consensus Conference (PEMVECC) recommends that all ventilated children should have their peripheral oxygen saturation (SpO_2_) monitored using pulse oximetry, and that patients with moderate to severe conditions should have their partial arterial oxygen pressure (PaO2) measured (1). This is done in order to prevent hypoxemia and hyperoxemia. On the other hand, this may need the manual adjustment of inspired oxygen, which may be an annoyance during time periods of high demand, such as the current epidemic of COVID-19. Furthermore, the pandemic has brought to light the need of increasing oxygen utilization in hospitals. This is due to the fact that it is probable that oxygen might become a scarce resource during times of such high demand (2, 3). The monitoring of SpO_2_ is the fundamental metric that is used to guide the treatment of acute respiratory failure in patients of all ages, including neonates, children, and adults. All of the recommendations that are now in place include specific sections on SpO_2_ monitoring, as well as ranges that are often imprecise but are up for debate based on the severity of the condition and the patient's age (1, 4, 5).

Both hypoxemia and hyperoxemia are conditions that pediatric intensivists often want to steer clear of (6–9). This precaution is rooted in previous research data has shown that there is a connection between excess or insufficient oxygen utilization and mortality in pediatric intensive care unit patients who have received oxygen treatment (10–14). Although the partial pressure of oxygen in the arterial system (PaO_2_) and the saturation of arterial oxygen (SaO_2_) are usually the measures that are used in the process of titrating oxygen, it is sometimes challenging to keep track of these values in pediatric patients. Pulse oximetry, often known as SpO_2_, is a potentially appealing option since it provides the benefit of ongoing tracking.

On November 29, 2023, a search was conducted in Embase, MEDLINE, CINAHL, and Web of Science using the keywords “closed-loop” or “automatic” and “oxygen” or “oxygen therapy.” There were no constraints placed on the search based on the publication date or language. The search resulted in the identification of 45 clinical investigations, of which 38 were randomized clinical trials. According to the findings of all of the investigations, SpO_2_ may be used by closed-loop oxygen systems in order to automatically modify the FiO_2_. The majority of these studies were conducted in neonates, with the remaining focus on adults; however, only two studies were carried out in pediatric patients. None of these research, on the other hand, investigated the effects of closed-loop oxygen regulation in pediatric patients while they were undergoing different modes of mechanical ventilation (15–56).

There is a current gap in research evaluating the effectiveness and safety of closed-loop oxygen systems in pediatric patients undergoing invasive mechanical ventilation for acute hypoxemic respiratory failure (AHRF), regardless of the applied ventilation mode. To address this gap, we conducted a randomized crossover study aimed at assessing the performance of a closed-loop oxygen control system integrated into a mechanical ventilator concerning the quality of oxygen therapy in pediatric patients. Our investigation also encompassed an evaluation of safety, determination of total oxygen consumption, and a comparison of manual adjustments between closed-loop oxygen control and manual oxygen titration. Our hypothesis postulated that the utilization of this closed-loop oxygen system would result in an increased duration within predefined optimal SpO_2_ ranges.

## Methods

### Study design

This study adopts a multicentre, single-blinded, randomized, crossover design to compare closed-loop oxygen control with manual oxygen titrations in the pediatric patient population across four medical facilities in Turkey. The eligible participants were carefully screened for inclusion in the PICUs at Dr Behcet Uz Children's Research and Training Hospital in Izmir, Aydin Obstetrics and Children Hospital in Aydin, Erzurum Territorial Training and Research Hospital in Erzurum, and Cam Sakura Research and Training Hospital in Istanbul. The enrolment period spanned from June 2022 to October 2022. Ethical approval was obtained from the Institutional Ethical Committee (Approval ID: 750/2022/29-09), and the study adhered to the principles outlined in the Declaration of Helsinki. Registration details for this study can be found on ClinicalTrials.gov (study identifier NCT05714527). Also the protocol including statistical plan was published online (57).

### Participants

Patients were eligible if they were (1) aged 1 month to 18 years and (2) receiving IMV with FiO_2_ > 25% to maintain SpO_2_ within clinician-defined parameters. (3) We excluded patients who had diseases or conditions that could potentially impact the measurement of transcutaneous SpO_2_, such as chronic or acute dyshemoglobinemia (including methemoglobinemia), carbon monoxide (CO) poisoning, and sickle cell disease. Additionally, we excluded patients who required a continuous infusion of epinephrine or norepinephrine at rates exceeding 1 mg/h. The exclusion criteria for this study included patients who had an immediate need for non-invasive ventilation or high-flow oxygen therapy whether foreseeable or unforeseeable, those with poor quality SpO_2_ measurements using finger and ear sensors, individuals with severe acidosis, pregnant women, patients at high risk for needing non-invasive mechanical ventilation or transportation to another unit or hospital, those with a formalized ethical decision to withhold or withdraw life support, patients participating in another research study, and patients who had previously been enrolled in the study during a previous episode of acute respiratory failure. The establishment of these criteria was aimed at guaranteeing the study's integrity and dependability, as well as the safety and wellbeing of the participants.

### Randomization and masking

Participants who were already intubated and receiving IMV due to the natural course and treatment of their disease, were assigned randomly to either begin with a 2-h session of closed-loop oxygen control or a 2-h session of manual oxygen titration. Subsequently, patients were transitioned to the alternative treatment. The randomization was conducted in a 1:1 ratio, using blocks of 4 and sealed opaque envelopes. The intervention design precluded the possibility of blinding healthcare professionals. Nonetheless, patients remained blinded about the specific techniques employed to regulate their oxygen levels.

### Procedures

The patients underwent intubation using an endotracheal tube of appropriate size, which was inserted properly. Throughout the trial, the patients were maintained in a semi–recumbent position. Invasive mechanical ventilation was performed using a pediatric ventilator which included a closed-loop oxygen controller (Hamilton–C1 or C6, Hamilton Medical AG, Bonaduz, Switzerland). Patients were administered sedatives as required, achieving an appropriate amount of sedation for each individual. The amount of sedation-analgesia remained constant during the whole course of the study. Continuous patient care and routine tasks, such as suctioning secretions or providing nutrition, were carried out without interruption, and randomly throughout both periods. At the research locations, the doctor to patient ratios during daytime and night-time shifts were roughly 6:1 and 12:1, respectively. Similarly, the nurse to patient ratios were around 2:1 and 3:1 during daytime and night-time shifts, respectively. The overall setting remained equivalent throughout the trial, meaning that these ratios did not alter between the two crossover periods. Furthermore, there was an absence of additional research staff throughout these two phases.

Following randomization, the attending pediatric intensivist determined the optimum range of oxygen saturation (SpO_2_) for each patient, taking into account their specific clinical condition and medical background. The term “optimal SpO_2_ target” does not represent a universal optimal for all patients. Instead, the optimal range is the ideal SpO_2_ level tailored to the patient's particular condition, taking into account factors such as lung compliance, driving pressure (ΔP), plateau pressure (Pplat), and positive end-expiratory pressure (PEEP). This approach ensures sufficient oxygenation while mitigating the risks associated with excessively high or low oxygen levels. Prior to moving on to the second 2-h session using the alternative oxygen titration approach, a 30-min washout interval was instituted after the first 2 h with the initial oxygen titration approach (Supplementary Figure 1). By using closed-loop oxygen control, the patients' SpO_2_ levels were maintained within a predetermined target range by the automated adjustment of the FiO_2_. During the manual oxygen titration phase of the trial, healthcare professionals at the bedside made manual changes to the FiO_2_, while maintaining the same target range for SpO_2_. For the two crossover stages of the study, the ventilation settings were unchanged. The SpO_2_ target range was established by determining four thresholds: an upper and lower threshold for the “optimum” range, and an upper and lower threshold for the “suboptimal” range. The optimum thresholds ranged from 94% to 98%, 93% to 97%, 92% to 96%, or 88% to 92%. The respective suboptimal thresholds were reported in Supplementary Table 1. The operational concepts of the closed-loop control are elaborated in Supplementary Table 2 and in the protocol paper (57).

### Data collection

Case report forms (CRFs) were used to record clinical and epidemiologic data. Using the ventilator's RS-232 interface connector, a Memory Box (Hamilton Medical AG) was attached to record ventilation data, including FiO_2_, SpO_2_, waveforms, alarms, and manual titrations, breath by breath. Patients' SpO_2_ was meticulously monitored using a Masimo Set sensor, specifically the Masimo RD model (Masimo Corp., Irvine, CA, USA), attached to their finger. This sensor provided the signal utilized by the closed-loop controller to ensure precise and automated FiO_2_ adjustments.

### Definitions

Each SpO_2_ reading was categorized as either optimal if it fell within the patient's predetermined range, suboptimal high or low if it fell outside of the optimal range but still within the suboptimal cut-offs, or unacceptable if it fell outside of the suboptimal range (Supplementary Table 1).

### Outcomes

The main aim of the research was to evaluate the efficacy of closed-loop oxygen control in the context of invasive ventilation for pediatric patients. Hence, the main objective was to determine the percentage of time spent within certain predetermined SpO_2_ goal ranges throughout each 2-h interval. The secondary outcomes included the percentage of time spent in suboptimal and unacceptable SpO_2_ ranges, the FiO_2_ and SpO_2_/FiO_2_ ratio, the frequency of manual oxygen changes, and the number of alarms.

The goal of the research was to assess the effectiveness of closed-loop oxygen control in the context of invasive ventilation for pediatric patients. Hence, we selected the primary objective to ascertain the proportion of time spent within predetermined SpO_2_ goal ranges during each 2-h interval. Secondary outcomes encompassed the percentage of time spent in suboptimal and unacceptable SpO_2_ ranges, the FiO_2_ and PaO2/FiO_2_ ratio, the frequency of manual oxygen adjustments, and the number of alarms. Furthermore, we evaluated the percentage of time with an available SpO_2_ signal to gauge the reliability of the oxygen monitoring system. Additionally, the study examined total oxygen consumption to provide insights into overall oxygen utilization during the research period.

### Power calculation

The determination of the sample size was based on data recorded from the pilot study involving seven patients (7 × 4 h = 28 h). This initial investigation aimed to assess the same disparity in the percentage of time allocated to optimal SpO_2_ target ranges during closed-loop vs. manual oxygen titrations. Utilizing the data from this pilot study, G^*^Power analysis indicated the necessity of including an additional 32 patients to achieve a statistical power of 95% (with a two-tailed α of 0.05) for detecting an effect size of Cohen's d = 0.68 in a Wilcoxon signed–rank test (58).

To accommodate potential dropouts, defined as instances where patients required extubation or noninvasive ventilation support, withdrew consent, experienced poor SpO_2_ readings for over 1 h during either study phase, or encountered technical recording issues, we opted for a final sample size of 35 patients.

### Statistical analysis

The normality of data distribution was assessed using Shapiro–Wilk, skewness, and kurtosis tests. Continuous data were presented as mean and standard deviation (SD) or median and interquartile range [IQR], depending on the nature of the distribution.

Statistical analysis involved the use of either a paired samples *t*-test or Wilcoxon test, selected based on appropriateness for the data. Specifically, the Wilcoxon signed–rank test was employed to compare the percentage of time spent within the target SpO_2_ range with manual FiO_2_ adjustments against the percentage with closed–loop FiO_2_ control.

A significance level of <0.05 was deemed statistically meaningful for all comparisons. Data were processed using MATLAB (version 2021b) by The MathWorks, Inc., Natick, Massachusetts, United States, while XLSTAT (version 2016) by Addinsoft, Paris, France, was utilized for statistical testing. Visual representations were generated using JASP (version 2022) by the JASP Team, Amsterdam, The Netherlands, and GraphPad PRISM (version 9) in San Diego, California, USA.

## Results

**Figure 1:**
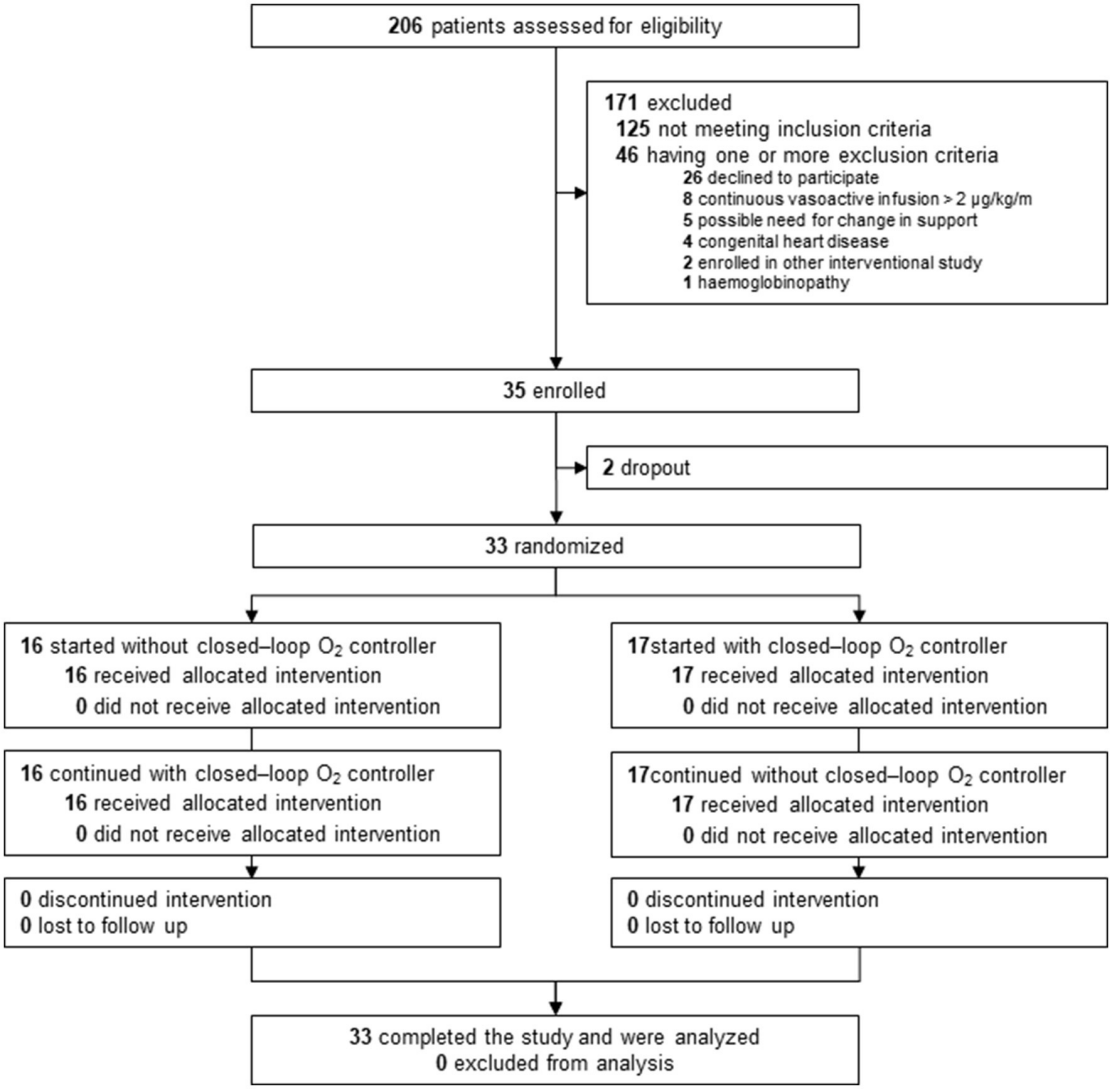
Trial profile.

Between June 2023 and December 2023, a total of 206 patients underwent screening. Out of these, 81 patients were found to be eligible, but 46 of them were excluded due to meeting one or more exclusion criteria. Ultimately, 35 patients were included in the study, with 2 patients dropping out. Therefore, a total of 33 patients were analyzed (Figure 1). Table 1 displays basic characteristics. The majority of patients were under 1.5 years old, weighed <15 kilograms, and in nearly half of the cases, AHRF was caused by a respiratory infection.

**Table 1:** Baseline characteristics of the study cohort.

**Variables**	**Median (IQR 25–75) or mean (SD) or n (%)**
Gender ratio (%f/%m)	43/57
Age (months)	17 (13–55.5)
IBW (kg)	13 (10.3–20)
PIM3	10.5 (0.8–20.6)
PELOD	12.9 (1–32.7)
PICU duration (days)	18 (8–22.5)
PEEP (cmH_2_O)	7 (5–8)
PIP (cmH_2_O)	25.3 (19.9–33.1)
**Admission diagnosis**	
Respiratory *A.pneumonia* *A.bronchiolitis* *Cystic fibrosis*	15 (45)
Sepsis	8 (24)
Neurologic *SE* *Meningoencephalitis*	5 (15)
Renal/Metabolic *RTA* *DKA*	3 (9)
Cardiovascular *VSD* *PDA*	2 (7)
**Lung physiology**	
Obstructive	5 (15)
Restrictive	10 (30)
Mixed	18 (55)
**Ventilation Mode**	
APV-SIMV	19 (57)
P-SIMV	8 (24)
ASV	5 (15)
SPONT	1 (3)

Data are expressed as median (interquartile range, IQR) or as mean (standard deviation, SD) or number and percentage. IBW, Ideal body weight; PIM3, Pediatric index of mortality 3, probability of death; PELOD, Pediatric logistic organ dysfunction, probability of death; *A.pneumonia*, Acute pneumonia; *A.bronchiolitis*, Acute bronchiolitis; SE, Status epilepticus; RTA, Renal tubular acidosis; DKA, Diabetic Keto Acidosis; VSD, Ventricular septal defect; PDA, Patent ductus arteriosus; SIMV, Synchronized intermittent mandatory ventilation ASV; APV, Adaptive Pressure Ventilation; ASV, Adaptive support ventilation; SPONT, Spontaneous ventilation mode; PIM3, Pediatric Index of Mortality; PELOD, Pediatric Logistic Organ Dysfunction; PICU, Pediatric Intensive Care Unit; PEEP, Positive End-Expiratory Pressure; PIP, Peak Inspiratory Pressure.

**Figure 2:**
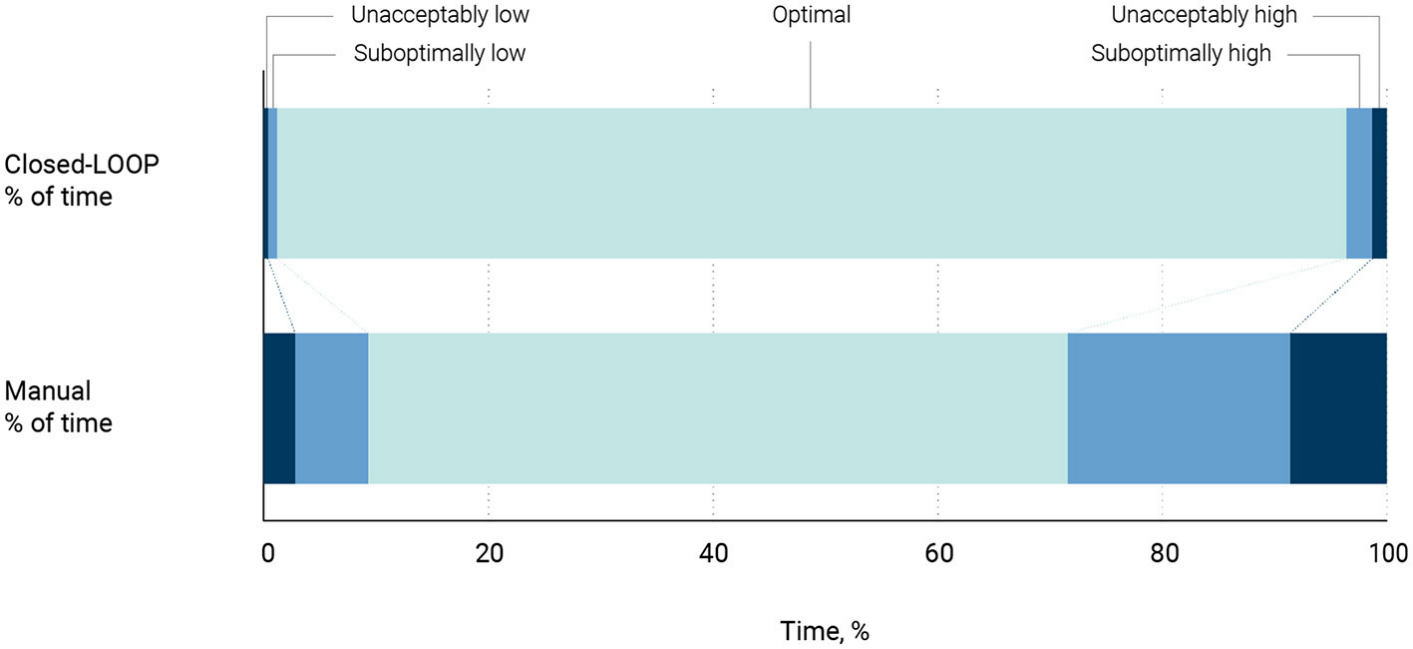
Time spent in optimal, suboptimal and unacceptable SpO_2_ ranges.

**Figure 3:**
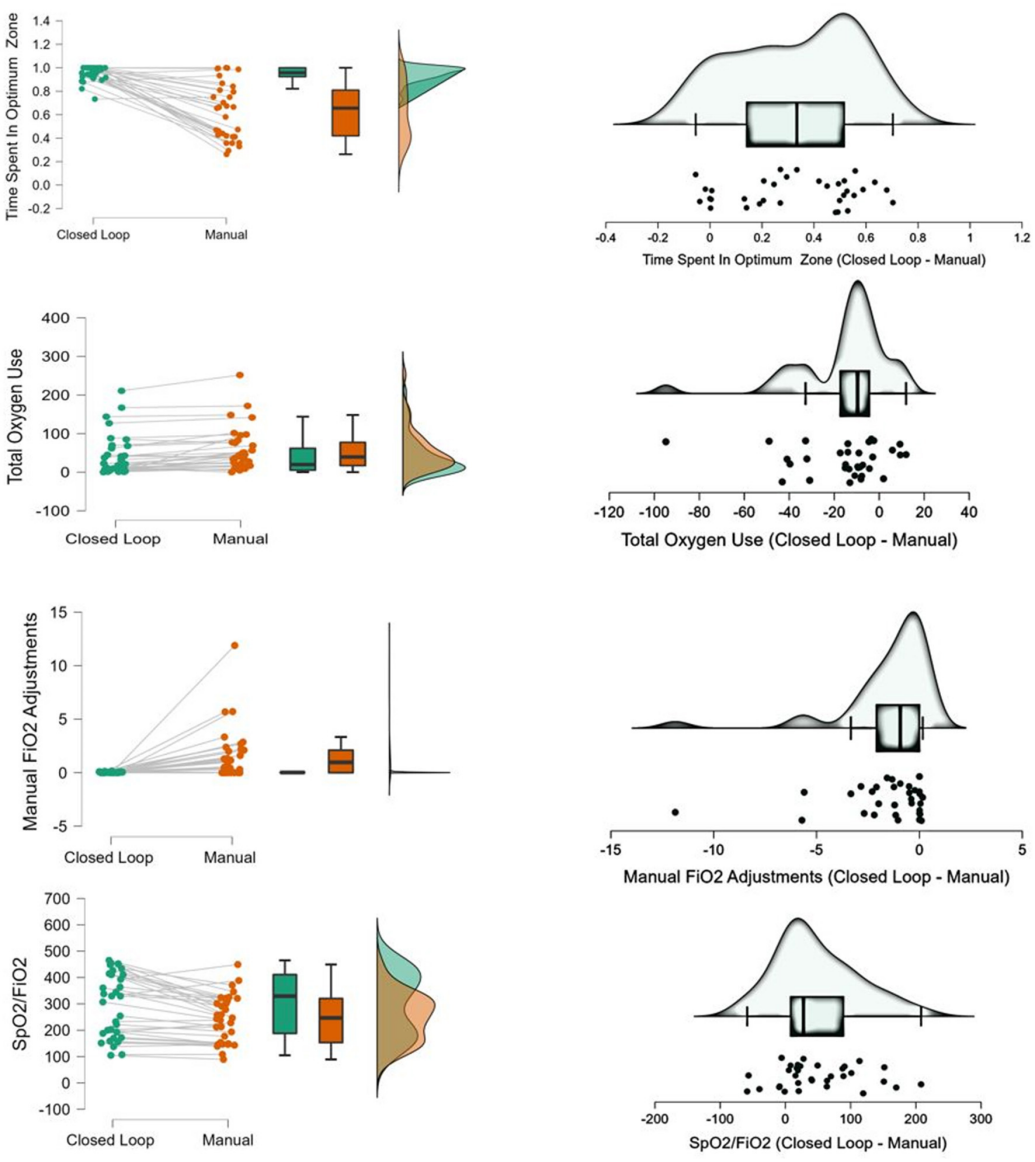
Effect of closed-loop control on time spent in optimum SpO_2_ zone, total oxygen use, manual FiO_2_ adjustments and SpO_2_/FiO_2._ The **left** panels collectively illustrate the effectiveness of the closed-loop system compared to manual adjustments across several parameters. The time spent in the optimum SpO_2_ zone is generally higher and more consistent with the closed-loop system, as indicated by the individual data points and box plots. Additionally, total oxygen use is lower with the closed-loop system, reflecting its efficiency in oxygen utilization. The need for manual FiO_2_ adjustments is significantly reduced when using the closed-loop system, highlighting its automation advantage. Furthermore, the SpO_2_/FiO_2_ ratio is higher and more stable with the closed-loop system, demonstrating better oxygenation efficiency. These findings suggest that the closed-loop system provides superior control and management of oxygen levels in patients. The **right** panels depict the differences between the closed-loop and manual methods for each parameter through density plots and scatter plots. For time spent in the optimum SpO_2_ zone, the closed-loop system shows a higher and more consistent distribution compared to manual adjustments. The total oxygen use is clearly lower with the closed-loop system, indicating its greater efficiency. The need for manual FiO_2_ adjustments is considerably fewer with the closed-loop system, as shown by the shift toward fewer adjustments. Lastly, the SpO_2_/FiO_2_ ratio is maintained at a higher level with the closed-loop system, reflecting better overall oxygenation. These visualizations confirm the advantages of the closed-loop system in providing efficient and effective oxygen management.

When the oxygen controller was enabled, patients spent a significantly higher amount of time within the ideal SpO_2_ ranges compared to manual oxygen titrations (95.7% [IQR 92.1%−100%] vs. 65.6% [IQR 41.6%−82.5%], mean difference 33.4% [95%–CI 24.5 to 42]); *P* < 0.001) (Table 2; Figures 2, 3).

**Table 2:** Primary and secondary outcomes.

**Variable**	**Closed-Loop**	**Manual**	**Median difference (95%CI)**	***P*-value**
**Primary outcome**				
Time spent in optimal SpO_2_ range (%)	95.7 (92.1 to 100)	65.6 (41.6 to 82.5)	33.4 (24.5 to 42)	<0.001
**Secondary outcomes**				
Time spent in suboptimal SpO_2_ range (%)				
Low	0.2 (0 to 1.2)	0.3 (0 to 5.6)	−1.7(−10.6 to 0.1)	0.147
High	0.3 (0 to 3.6)	14.2 (2.3 to 31.4)	−18.6 (−27.3 to −11.4)	0.001
Total	1.7 (0 to 5.1)	27.2 (10.3 to 39.5)	−22.8 (−29.4 to −15.8)	<0.001
Mean FiO_2_ (%)	32.1 (23.9 to 54.1)	40.6 (31.1 to 62.8)	−6 (−8 to −3.9)	<0.001
Mean SpO_2_/FiO_2_	329.4 (180 to 411.1)	246.7 (151.1 to 320.5)	44.5 (20 to 69.8)	<0.001
Manual Adjustments (n/h)	0 (0 to 0)	1 (0 to 2.2)	−1.7 (−3 to −1.2)	<0.001
Alarms (n/h)	0 (1 to 1.3)	0 (0 to 0.8)	−0.3 (−3.8 to 2.5)	0.69
Percentage of time SpO_2_ available	99.9 (99.3 to 100)	99.9 (98.7 to 100)	0.8 (−0.003 to 3.4)	0.05
Percentage of time SpO_2_ <88%	0 (0 to 0.2)	0 (0 to 0.4)	−0.3 (−1.8 to 0.1)	0.22
Percentage of time SpO_2_ <85%	0 (0 to 0.07)	0 (0 to 0.2)	−0.4 (−0.9 to −0.06)	0.02
Number of events SpO_2_ <88%	0 (0 to 0.4)	0 (0 to 0.5)	−0.2 (−0.9 to 0.4)	0.29
Percentage of time FiO_2_ <40%	83.9 (0 to 1)	48.6 (0 to 99.9)	15.9 (−1.6 to 36)	0.07
Percentage of time 40% ≤ FiO_2_ ≤ 60%	0.5 (0 to 60.2)	0.7 (0 to 64.9)	1.3 (−25.5 to 16.5)	0.94
Percentage of time FiO_2_ >60%	0 (0 to 10.9)	0 (0 to 35.1)	−5.6 (−30.7 to −0.01)	0.05
Total Oxygen Use (L/h)	19.8 (4.6 to 64.8)	39.4 (16.8 to 79.9)	−11.7 (−20.9 to −7.7)	<0.001

Data are expressed as median (interquartile range, IQR) or as mean (standard deviation, SD). Wilcoxon or student's *t* test were performed depending on each variable distribution according to the Shapiro–Wilk test. 95%CI, 95% confidence interval; SpO_2_, peripheral oxygen saturation; FiO_2_, Fraction of inspired oxygen.

Upon activating the oxygen controller, patients spent considerably less time in the total unacceptably and suboptimal SpO_2_ ranges (Table 2, Figure 2). Also, this activation significantly reduced the duration patients were exposed to SpO_2_ levels considered unacceptably high and sub-optimally high (Table 2, Figure 2).

The adoption of closed-loop oxygen controller led to a significant reduction in both the mean fraction of inspired oxygen (FiO_2_) and total oxygen utilization (Table 2). Moreover, the SpO_2_/FiO_2_ ratio was significantly increased under the closed-loop oxygen controller (Table 2; Figure 3). Closed-loop controller also markedly decreased the frequency of manual interventions required (Table 2; Figures 2, 3).

## Discussion

This multicenter randomized controlled crossover trial, focusing on pediatric patients treated with invasive mechanical ventilation for AHRF, reveals the following outcomes: The implementation of a closed-loop oxygen controller within a mechanical ventilator, as opposed to manual oxygen titration, brought about numerous significant benefits: The closed-loop system substantially increased the time that patients' SpO_2_ levels remained what we pre-defined as the optimal range. This precise regulation ensures that patients receive oxygen to maintain a reasonable range of oxygenation and avoid hypoxemia and hyperoxia. The closed-loop controller effectively reduced the periods during which patients' SpO_2_ levels were outside the physician predefined optimal range. This reduction in time spent at suboptimal levels means that patients are less likely to experience the adverse effects associated with inadequate or excessive oxygenation. The system's ability to swiftly respond to changes in the patient's condition ensures a higher level of care and reduces potential complications. One of the standout advantages of the closed-loop system is its efficiency in oxygen use. By delivering oxygen more precisely and only when necessary, the system reduces overall oxygen consumption. This not only has economic benefits but also lessens the burden on oxygen supply systems, making it particularly valuable in resource-limited settings. The meticulous management of FiO_2_ means that no excess oxygen is wasted, contributing to more sustainable healthcare practices. The closed-loop oxygen controller significantly lowered the need for manual adjustments by healthcare providers. This automation allows medical staff to focus more on other critical aspects of patient care, enhancing overall efficiency within the clinical environment. The reduction in manual intervention also means that there is less room for human error, thereby improving the safety and reliability of oxygen therapy. This feature is particularly beneficial in busy or understaffed medical settings, where it can greatly enhance the quality of patient management. In summary, the introduction of a closed-loop oxygen controller in mechanical ventilators offers a transformative approach to oxygen therapy, ensuring precise, efficient, and safe management of SpO_2_ levels. Its ability to maintain optimal oxygen saturation, reduce suboptimal periods, lower oxygen consumption, and minimize manual interventions makes it a superior alternative to traditional manual oxygen titration methods. Our research has several benefits, both conceptually and in terms of execution. We used a crossover approach to compare the effectiveness of closed-loop oxygen control and manual oxygen titrations for each participant, making our findings statistically more robust. We conducted the study across multiple centers, including universities and teaching hospitals, to increase the generalizability of our results. We adhered to a published protocol and used randomization mechanisms to minimize the risk of bias. Additionally, we established an analytic strategy before finalizing the database, which involved predefining optimal, suboptimal, and unacceptable SpO_2_ values based on previous consensus. This ensured the objectivity and reliability of our results. To the extent of our knowledge, this study is the inaugural effort to examine the effectiveness of closed-loop oxygen management in pediatric patients receiving invasive mechanical ventilation, regardless of ventilation mode reliance.

The results of our investigation are consistent with previous research studies that have evaluated the efficacy of closed-loop oxygen regulation in groups of premature infants, pediatric and adult patients undergoing either invasive or non-invasive mechanical ventilation for acute respiratory failure caused by various factors (15–56, 59–67).

Throughout these investigations, closed-loop oxygen control consistently exhibited superior efficacy compared to manual oxygen titration by healthcare personnel. This was demonstrated by improved adherence to target peripheral oxygen saturation (SpO_2_) ranges and decreased duration spent within potentially dangerous SpO_2_ levels. Our study enhances the current understanding of the effectiveness of closed-loop oxygen control in pediatric patients with acute hypoxemic respiratory failure (AHRF). It clarifies that closed-loop oxygen control is superior to manually adjusting the FiO_2_ in cases where there is clinical instability. This demonstrated superiority is particularly significant because to its potential to alleviate the onerous tasks carried by healthcare workers in the intensive care unit (ICU), whose workload is sometimes exacerbated by the need of patient stabilization (68, 69).

Our findings are also consistent with previous studies investigating closed-loop oxygen controllers in invasively ventilated neonates (19, 59–61). Similar results have been reported in the pediatric population (53) and adult patients (62–67). This body of research demonstrates the superiority of closed-loop oxygen control compared to manual titration methods in patients receiving respiratory support. Notably, oxygenation in these patients is influenced not only by FiO_2_, but also by factors such as delivered tidal volumes and airway pressures. Collectively, these findings suggest a broad applicability of closed-loop oxygen control for critically ill hypoxemic patients receiving various forms of respiratory support. This includes both passive and active breathers, too. Not all of our patients were under assisted mechanical ventilation; some were in a passive state. This variation in patient ventilation modes could be perceived as a limitation of our study, as it introduces a level of heterogeneity that might affect the generalizability of our findings. Additionally, the generalizability of our results is limited to similar settings, and this should be duly noted. It is widely recognized that medical professionals specializing in intensive care, including physicians and nurses, diligently prevent both hypoxemia and hyperoxemia due to numerous justifiable concerns. This approach is especially pertinent among healthcare providers who manage critically ill neonatal and pediatric patients (6–9). This strategy necessitates not only proficient health care providers but also a significant number of intensive care unit (ICU) staff directly attending to the patient. Minimizing hypoxemia can only be achieved if there is a consistent presence of a nurse who can do manual oxygen adjustments (59, 60). This condition can be deemed both unfeasible and costly, and may not be regularly fulfilled.

Recent investigations, including an older Canadian study and a more recent one from the Netherlands, have highlighted an asymmetrical approach by physicians in managing SpO_2_ levels beyond the optimal zones. While physicians strive to avert both hypoxemia and hyperoxemia, there is a greater focus on preventing hypoxemia. Consequently, this often leads to extended periods within suboptimal or high SpO_2_ ranges when manually adjusting oxygen levels (70, 71). In contrast, the implementation of a closed-loop oxygen control system in our experimental arm, demonstrates its efficacy by equally preventing deviations into both lower and higher SpO_2_ ranges, thus maintaining a more stable patient condition.

The observation that SpO_2_/FiO_2_ ratios were greater in the context of closed-loop oxygen control indicates that this method not only averts hypoxemic and hyperoxemic deterioration, but also enhances overall oxygenation. Concurrently, it achieves the same or better levels of oxygenation with reduced oxygen consumption, which might be crucial in contexts with limited resources or during times of increased demand, such as a pandemic. This finding aligns with several previous studies that have shown patients receiving closed-loop control of FiO_2_ got a lower quantity of FiO_2_ than those who underwent manual titration (51, 53, 60, 62, 63, 72). Moreover, the utility of automatizing oxygen delivery is especially significant in low- and middle-income countries (LMICs), where the lack of personnel and the high consumption of oxygen are prominent challenges. Automated systems can ensure consistent and precise oxygen delivery, thereby alleviating the burden on limited healthcare staff and conserving essential oxygen supplies. Even in high-resource countries, replacing the nursing workforce at the bedside has become increasingly difficult post-pandemic. The implementation of closed-loop oxygen controllers can therefore play a pivotal role in both high- and low-resource settings by optimizing oxygen use and reducing the dependency on manual titration, ultimately enhancing patient care and resource management.

We selected the same SpO_2_ target range for both the closed-loop group and the manual group. However, in the control arm, patients spent more time in the suboptimal high SpO_2_ range, indicating that the mean SpO_2_ was higher during the manual titration period. This discrepancy in SpO_2_/FiO_2_ ratios could potentially be attributed to denitrogenation atelectasis, as more than 25% of the patients in the manual arm were exposed to FiO_2_ levels exceeding 60% at some point during the study (73). Nevertheless, we lack concrete evidence to substantiate the occurrence of this type of atelectasis within our study group. Moreover, no significant changes were observed in driving pressure or tidal volume (TV) during the two phases of the study to support this hypothesis.

Surprisingly, we saw far fewer manual adjustments, yet a statistically significant reduction in the number of hourly alarms with closed-loop oxygen regulation. This may potentially result in decreased workloads, since our research indicates that the implementation of a closed-loop oxygen controller requires trained healthcare provider adjustments less than manual oxygen titration (74). Increased ICU staff workloads are correlated with higher mortality rates. Furthermore, our findings indicate a modification in the intensity of alarms toward a more tolerable level, thereby enhancing patient comfort and sleep hygiene while concurrently mitigating the likelihood of delirium (75, 76).

The study reported here has certain limitations that should be taken into account. Initially, the time allocated for both the manual and automated oxygen titration procedures was limited to a mere 2 h, which is not enough to cover the whole spectrum of everyday activities that patients go through. The restricted duration was selected to ensure uniformity in patient circumstances throughout both phases of the crossover trial, which is critical due to the fast fluctuations that may occur in pediatric patients. In addition, both stages were intentionally planned to be carried out inside a single shift, which unavoidably limits the thorough examination of each case within these time periods.

The crossover design of the study also restricts our ability to assess the effects of closed-loop oxygen control on pertinent clinical outcomes, such as the duration of invasive mechanical ventilation, the shift from invasive to non-invasive ventilation, and the process of gradually reducing respiratory support for patients. Furthermore, the nature of the intervention precluded the blinding of healthcare workers involved in the study. Nevertheless, in order to adhere to regular clinical procedures, predetermined SpO_2_ zones were used, mirroring the zones to which ICU nurses often adapt FiO_2_.

We did not include patients with skin types darker than Fitzpatrick scale 4 in this study. However, we acknowledge that there is a significant difference in melanin levels between Fitzpatrick skin types 1–2 and type 4. Consequently, it is possible that adjustments for skin pigmentation could be necessary even within the range of Fitzpatrick skin types included in our study. This represents a limitation of our study, as the continuum of melanin levels across the full Fitzpatrick scale, or more accurately, the Monk Skin Tone Scale, should be considered to better account for variations in skin pigmentation in future research It is important to note that pulse oximetry can overestimate true SaO_2_ in individuals with darker skin tones. While the clinical relevance of this bias remains unclear, its magnitude is likely to be more significant when SaO_2_ is lower (77). Consequently, future research should take into account skin color when defining personal optimal targets to ensure accuracy and efficacy in oxygen therapy across diverse patient populations.

Future research should prioritize investigating these clinical goals to improve comprehension of the consequences of closed-loop oxygen titration approaches in pediatric intensive and emergency care.

In many liberal vs. limited oxygen trials, such as the most recent OXY-PICU trial, researchers have struggled to maintain low SpO_2_ levels in the restricted arm. Although the restricted arm in the OXY-PICU study aimed for an SpO_2_ level of 88 to 92%, they could only achieve a median SpO_2_ of 94% (IQR 93–96) for the conservative group, compared to 97% (96–98) for the liberal oxygenation group (78). Therefore, closed-loop oxygen controllers may play a very important potential role in these types of studies in the future, ensuring more precise maintenance of target SpO_2_ levels. One significant limitation of our study is the use of the terms “optimal” and “ideal oxygenation” without sufficient nuance. Currently, there is insufficient data to definitively determine what constitutes ideal or optimal oxygenation. Furthermore, the impact of different oxygenation levels on clinical outcomes has not been evaluated in this study. Future research is needed to establish clear guidelines and evidence-based practices for optimal oxygenation to improve patient outcomes. Until such data are available, the terms “optimal” and “ideal” should be interpreted with caution, and our findings should be viewed as preliminary in this context.

In conclusion, when evaluating the efficacy of closed-loop oxygen control vs. manual oxygen titrations in pediatric patients undergoing invasive mechanical ventilation for AHRF, significant benefits are observed with the closed-loop system. Notably, it enhances the duration that patients remain within optimal SpO_2_ ranges and diminishes the periods spent in SpO_2_ zones that may pose risks. Furthermore, closed-loop oxygen control not only improves overall oxygenation but also conserves oxygen resources and reduces the necessity for manual adjustments. These findings suggest that implementing closed-loop systems could offer substantial clinical advantages in managing pediatric AHRF.

## Data Availability

The raw data supporting the conclusions of this article will be made available by the authors, without undue reservation.

## Supplementary material

Supplementary Figure 1 Trial flow diagram.

Supplementary Table 1 SpO_2_ predefined targets.

Supplementary Table 2 Principles of Closed-loop oxygen controller.

## References

[1] Kneyber MCJ de Luca D Calderini E Jarreau P-H Javouhey E Lopez-Herce J. Recommendations for mechanical ventilation of critically ill children from the Paediatric Mechanical Ventilation Consensus Conference (PEMVECC). Intensive Care Med. (2017) 43:1764–80. 10.1007/s00134-017-4920-z 28936698 PMC5717127

[2] Shrestha GS Lamsal R. Rational Use of Oxygen in COVID-19 Pandemic - Are We Doing Enough? JNMA J Nepal Med Assoc. (2021) 59:429–31. 10.31729/jnma.6479 34508518 PMC8369598

[3] Sindwani G Suri A. Acute hospital oxygen shortage during COVID-19 pandemic surge: how can we prevent the apocalypse? Brazilian J Anesthesiol. (2022) 72:311–2. 10.1016/j.bjane.2021.10.003 34793783 PMC8591984

[4] Group TPALICC. Pediatric acute respiratory distress syndrome: consensus recommendations from the pediatric acute lung injury consensus conference^*^. Pediatr Crit Care Med. (2015) 16:428–39. 10.1097/PCC.0000000000000350 25647235 PMC5253180

[5] Emeriaud G López-Fernández YM Iyer NP Bembea MM Agulnik A Barbaro RP. Executive summary of the second international guidelines for the diagnosis and management of pediatric acute respiratory distress syndrome (PALICC-2). Pediatr Crit Care Med. (2023) 24:143–68. 36661420 10.1097/PCC.0000000000003147 PMC9848214

[6] Singer M Young PJ Laffey JG Asfar P Taccone FS Skrifvars MB. Dangers of hyperoxia. Critical Care. (2021) 25:440. 10.1186/s13054-021-03815-y 34924022 PMC8686263

[7] Palmer E Post B Klapaukh R Marra G MacCallum NS Brealey D. The association between supraphysiologic arterial oxygen levels and mortality in critically ill patients. A multicenter observational cohort study. Am J Respir Crit Care Med. (2019) 200:1373–80. 10.1164/rccm.201904-0849OC 31513754 PMC6884048

[8] de Jonge E Peelen L Keijzers PJ Joore H de Lange D van der Voort PH. Association between administered oxygen, arterial partial oxygen pressure and mortality in mechanically ventilated intensive care unit patients. Crit Care. (2008) 12:R156. 10.1186/cc7150 19077208 PMC2646321

[9] Young PJ Hodgson CL Rasmussen BS. Oxygen targets. Intensive Care Med. (2022). 10.1007/s00134-022-06714-0 35511273

[10] Pelletier JH Ramgopal S Au AK Clark RSB Horvat CM. Maximum Pao2 in the first 72 hours of intensive care is associated with risk-adjusted mortality in pediatric patients undergoing mechanical ventilation. Crit Care Explor. (2020) 2:e0186. 10.1097/CCE.0000000000000186 32984827 PMC7491884

[11] Peters MJ Jones GA Wiley D Wulff J Ramnarayan P Ray S. Conservative versus liberal oxygenation targets in critically ill children: the randomised multiple-centre pilot Oxy-PICU trial. Intensive Care Med. (2018) 44:1240–8. 10.1007/s00134-018-5232-7 29868973

[12] Page D Ablordeppey E Wessman BT Mohr NM Trzeciak S Kollef MH. Emergency department hyperoxia is associated with increased mortality in mechanically ventilated patients: a cohort study. Crit Care. (2018) 22:9. 10.1186/s13054-017-1926-4 29347982 PMC5774130

[13] Stenson BJ Tarnow-Mordi WO Darlow BA Simes J Juszczak E Askie L. Oxygen saturation and outcomes in preterm infants. N Engl J Med. (2013) 368:2094–104. 23642047 10.1056/NEJMoa1302298

[14] Lilien TA Groeneveld NS van Etten-Jamaludin F Peters MJ Buysse CM Ralston SL. Association of arterial hyperoxia with outcomes in critically ill children: a systematic review and meta-analysis. JAMA Netw Open. (2022) 5:e2142105. 10.1001/jamanetworkopen.2021.42105 34985516 PMC8733830

[15] Mitra S Singh B El-Naggar W McMillan DD. Automated versus manual control of inspired oxygen to target oxygen saturation in preterm infants: a systematic review and meta-analysis. J Perinatol. (2018) 38:351–60. 10.1038/s41372-017-0037-z 29296004

[16] Reynolds PR Miller TL Volakis LI Holland N Dungan GC Roehr CC. Randomised cross-over study of automated oxygen control for preterm infants receiving nasal high flow. Arch Dis Child Fetal Neonatal Ed. (2019) 104:F366–f71. 10.1136/archdischild-2018-315342 30464005

[17] Waitz M Schmid MB Fuchs H Mendler MR Dreyhaupt J Hummler HD. Effects of automated adjustment of the inspired oxygen on fluctuations of arterial and regional cerebral tissue oxygenation in preterm infants with frequent desaturations. J Pediatr. (2015) 166:240–4.e1. 10.1016/j.jpeds.2014.10.007 25454938

[18] Bourassa S Bouchard P-A Dauphin M Lellouche F. Oxygen conservation methods with automated titration. Respir Care. (2020) 65:1433–42. 10.4187/respcare.07240 32071135

[19] Kaam AH Hummler HD Wilinska M Swietlinski J Lal MK Pas AB. Automated versus manual oxygen control with different saturation targets and modes of respiratory support in preterm infants. J Pediatr. (2015) 167:545–50. 10.1016/j.jpeds.2015.06.012 26144575

[20] Lal M Tin W Sinha S. Automated control of inspired oxygen in ventilated preterm infants: crossover physiological study. Acta Paediatr. (2015) 104:1084–9. 10.1111/apa.13137 26194933

[21] Dijkman KP Mohns T Dieleman JP van Pul C Goos TG Reiss IK. Predictive Intelligent Control of Oxygenation (PRICO) in preterm infants on high flow nasal cannula support: a randomised cross-over study. Arch Dis Childh Fetal Neonatal. (2021) 106:621–6. 10.1136/archdischild-2020-320728 33972265

[22] Dani C Pratesi S Luzzati M Petrolini C Montano S Remaschi G. Cerebral and splanchnic oxygenation during automated control of inspired oxygen (FiO2) in preterm infants. Pediatr Pulmonol. (2021) 56:2067–72. 10.1002/ppul.25379 33773084

[23] Das A Mhanna M Teleron-Khorshad A Houdek J Kumar N Gunzler D. A comparison of manual versus automated saturation of peripheral oxygenation in the neonatal intensive care unit. J Matern Fetal Neonatal Med. (2016) 29:1631–5. 10.3109/14767058.2015.1057493 26103781

[24] Dargaville PA Fathabadi OS Plottier GK Lim K Wheeler KI Jayakar R. Development and preclinical testing of an adaptive algorithm for automated control of inspired oxygen in the preterm infant. Arch Dis Child Fetal Neonatal Ed. (2017) 102:F31–f6. 10.1136/archdischild-2016-310650 27634820

[25] Zapata J Gómez JJ Araque Campo R Matiz Rubio A Sola A. A randomised controlled trial of an automated oxygen delivery algorithm for preterm neonates receiving supplemental oxygen without mechanical ventilation. Acta Paediatr. (2014) 103:928–33. 10.1111/apa.12684 24813808 PMC4228757

[26] Van Zanten HA Kuypers K Stenson BJ Bachman TE Pauws SC Te Pas AB. The effect of implementing an automated oxygen control on oxygen saturation in preterm infants. Arch Dis Child Fetal Neonatal Ed. (2017) 102:F395–f9. 10.1136/archdischild-2016-312172 28209638

[27] Sturrock S Ambulkar H Williams EE Sweeney S Bednarczuk NF Dassios T. A randomised crossover trial of closed loop automated oxygen control in preterm, ventilated infants. Acta Paediatr. (2021) 110:833–7. 10.1111/apa.15585 32969040

[28] Kaltsogianni O Dassios T Greenough A. Does closed-loop automated oxygen control reduce the duration of mechanical ventilation? A randomised controlled trial in ventilated preterm infants. Trials. (2022) 23:276. 10.1186/s13063-022-06222-y 35395952 PMC8994422

[29] Morozoff E Smyth JA Saif M. Applying computer models to realize closed-loop neonatal oxygen therapy. Anesth Analg. (2017) 124:95–103. 10.1213/ANE.0000000000001367 27992386

[30] Gajdos M Waitz M Mendler MR Braun W Hummler H. Effects of a new device for automated closed loop control of inspired oxygen concentration on fluctuations of arterial and different regional organ tissue oxygen saturations in preterm infants. Arch Dis Child Fetal Neonatal Ed. (2019) 104:F360–f5. 10.1136/archdischild-2018-314769 30154236

[31] Schwarz CE Kidszun A Bieder NS Franz AR König J Mildenberger E. Is faster better? A randomised crossover study comparing algorithms for closed-loop automatic oxygen control. Arch Dis Child Fetal Neonatal Ed. (2020) 105:369–74. 10.1136/archdischild-2019-317029 31527093

[32] Dargaville PA Marshall AP Ladlow OJ Bannink C Jayakar R Eastwood-Sutherland C. Automated control of oxygen titration in preterm infants on non-invasive respiratory support. Arch Dis Child Fetal Neonatal Ed. (2022) 107:39–44. 10.1136/archdischild-2020-321538 33963005

[33] Ali SK Jayakar RV Marshall AP Gale TJ Dargaville PA. Preliminary study of automated oxygen titration at birth for preterm infants. Arch Dis Child Fetal Neonatal Ed. (2022). 10.1136/archdischild-2021-323486 35140115

[34] Plottier GK Wheeler KI Ali SKM Fathabadi OS Jayakar R Gale TJ. Clinical evaluation of a novel adaptive algorithm for automated control of oxygen therapy in preterm infants on non-invasive respiratory support. Arch Dis Child Fetal Neonatal Ed. (2017) 102:F37–f43. 10.1136/archdischild-2016-310647 27573518

[35] Salverda HH Cramer SJ Witlox RS Gale TJ Dargaville PA Pauws SC. Comparison of two devices for automated oxygen control in preterm infants: a randomised crossover trial. Arch Dis Child Fetal Neonatal Ed. (2022) 107:20–5. 10.1136/archdischild-2020-321387 34112721 PMC8685610

[36] Schwarz CE Kreutzer KB Langanky L Wolf NS Braun W O'Sullivan MP. Randomised crossover trial comparing algorithms and averaging times for automatic oxygen control in preterm infants. Arch Dis Child Fetal Neonatal Ed. (2021) 107:425–30. 10.1136/archdischild-2021-322096 34819347

[37] Roca O Caritg O Santafé M Ramos FJ Pacheco A García-de-Acilu M. Closed-loop oxygen control improves oxygen therapy in acute hypoxemic respiratory failure patients under high flow nasal oxygen: a randomized cross-over study (the HILOOP study). Crit Care. (2022) 26:108. 10.1186/s13054-022-03970-w 35422002 PMC9008383

[38] L'Her E Jaber S Verzilli D Jacob C Huiban B Futier E. Automated closed-loop <em>versus </em> standard manual oxygen administration after major abdominal or thoracic surgery: an international multicentre randomised controlled study. Eur Respir J. (2021) 57:2000182. 10.1183/13993003.00182-2020 32855218

[39] Harper JC Kearns NA Maijers I Bird GE Braithwaite I Shortt NP. Closed-loop oxygen control using a novel nasal high-flow device: a randomized crossover trial. Respir Care. (2021) 66:416–24. 10.4187/respcare.08087 33082219

[40] Kofod LM Westerdahl E Kristensen MT Brocki BC Ringbæk T Hansen EF. Effect of automated oxygen titration during walking on dyspnea and endurance in chronic hypoxemic patients with COPD: a randomized crossover trial. J Clin Med. (2021) 10:4820. 10.3390/jcm10214820 34768338 PMC8584500

[41] Malli F Boutlas S Lioufas N Gourgoulianis KI. Automated oxygen delivery in hospitalized patients with acute respiratory failure: a pilot study. Canadian Respir J. (2019) 2019:4901049. 10.1155/2019/4901049 30863468 PMC6377968

[42] Lellouche F L'Her E. Automated oxygen flow titration to maintain constant oxygenation. Respir Care. (2012) 57:1254–62. 10.4187/respcare.01343 22348812

[43] Hansen EF Hove JD Bech CS Jensen JS Kallemose T Vestbo J. Automated oxygen control with O2matic(^®^) during admission with exacerbation of COPD. Int J Chron Obstruct Pulmon Dis. (2018) 13:3997–4003. 10.2147/COPD.S183762 30587955 PMC6300382

[44] Lellouche F Bouchard PA Roberge M Simard S L'Her E Maltais F. Automated oxygen titration and weaning with FreeO2 in patients with acute exacerbation of COPD: a pilot randomized trial. Int J Chron Obstruct Pulmon Dis. (2016) 11:1983–90. 10.2147/COPD.S112820 27601891 PMC5003517

[45] Poder TG Kouakou CR Bouchard PA Tremblay V Blais S Maltais F. Cost-effectiveness of FreeO(2) in patients with chronic obstructive pulmonary disease hospitalised for acute exacerbations: analysis of a pilot study in Quebec. BMJ Open. (2018) 8:e018835. 10.1136/bmjopen-2017-018835 29362258 PMC5786115

[46] Cirio S Nava S. Pilot study of a new device to titrate oxygen flow in hypoxic patients on long-term oxygen therapy. Respir Care. (2011) 56:429–34. 10.4187/respcare.00983 21255511

[47] Trottier M Bouchard PA L'Her E Lellouche F. Automated oxygen titration during CPAP and noninvasive ventilation in healthy subjects with induced hypoxemia. Respir Care. (2023) 68:1553–60. 10.4187/respcare.09866 37311626 PMC10589107

[48] Sandau C Hansen EF Ringbæk TJ Kallemose T Bove DG Poulsen I. Automated oxygen administration alleviates dyspnea in patients admitted with acute exacerbation of COPD: a randomized controlled trial. Int J Chron Obstruct Pulmon Dis. (2023) 18:599–614. 10.2147/COPD.S397782 37096159 PMC10122478

[49] Kaltsogianni O Dassios T Jenkinson A Greenough A. Does closed-loop automated oxygen control reduce the duration of supplementary oxygen treatment and the amount of time spent in hyperoxia? A randomised controlled trial in ventilated infants born at or near term. Trials. (2023) 24:404. 10.1186/s13063-023-07415-9 37316885 PMC10268377

[50] Kaltsogianni O Dassios T Harris C Jenkinson A Lee RA Sugino M. Closed-loop oxygen system in late preterm/term, ventilated infants with different severities of respiratory disease. Acta Paediatr. (2023) 112:1185–9. 10.1111/apa.16678 36656138

[51] Soydan E Ceylan G Topal S Hepduman P Atakul G Colak M. Automated closed–loop FiO2 titration increases the percentage of time spent in optimal zones of oxygen saturation in pediatric patients–A randomized crossover clinical trial. Front. Med. (2022) 9:969218. 10.3389/fmed.2022.969218 36091711 PMC9452913

[52] Sandau C Poulsen I Nørholm V Hansen EF Ringbaek TJ Suppli Ulrik C. Patients' perspective on automated oxygen administration during hospitalization for acute exacerbation of chronic obstructive pulmonary disease: a qualitative study nested in a randomized controlled trial. COPD. (2022) 19:345–52. 10.1080/15412555.2022.2141620 36416665

[53] Sandal O Ceylan G Topal S Hepduman P Colak M Novotni D. Closed–loop oxygen control improves oxygenation in pediatric patients under high–flow nasal oxygen—A randomized crossover study. Front Med. (2022) 9:1046902. 10.3389/fmed.2022.1046902 36465920 PMC9708705

[54] Salverda HH Beelen DML Cramer SJE Pauws SC Schalij-Delfos N Te Pas AB. Clinical outcomes of preterm infants while using automated controllers during standard care: comparison of cohorts with different automated titration strategies. Arch Dis Child Fetal Neonatal Ed. (2022) 108:26–30. 10.1136/archdischild-2021-323690 35577567

[55] Nair V Lal MK Gillone J Kannan Loganathan P Bachman TE. Comparison of volume guarantee and volume-controlled ventilation both using closed loop inspired oxygen in preterm infants: a randomised crossover study (CLIO-VG study). Arch Dis Child Fetal Neonatal Ed. (2022) 107:161–5. 10.1136/archdischild-2021-321712 34233907

[56] Kaltsogianni O Dassios T Belbal R Greenough A. Survey of closed-loop automated oxygen control systems in neonatal intensive care units. Acta Paediatr. (2022) 111:1002–3. 10.1111/apa.16239 34964169 PMC9303327

[57] Atakul G Ceylan G Sandal O Soydan E Topal S Colak M. Closed-loop O2 use during invasive mechanical ventilation of pediatric patients (CLOUDIMPP): a randomized cross-over study. Res Square. (2023) 1:2908224. 10.21203/rs.3.rs-2908224/v1 PMC11420134 39318593

[58] Faul F Erdfelder E Lang A-G Buchner A. G^*^Power 3: a flexible statistical power analysis program for the social, behavioral, and biomedical sciences. Behav Res Methods. (2007) 39:175–91. 10.3758/BF03193146 17695343

[59] Claure N Gerhardt T Everett R Musante G Herrera C Bancalari E. Closed-loop controlled inspired oxygen concentration for mechanically ventilated very low birth weight infants with frequent episodes of hypoxemia. Pediatrics. (2001) 107:1120–4. 10.1542/peds.107.5.1120 11331696

[60] Claure N D'Ugard C Bancalari E. Automated adjustment of inspired oxygen in preterm infants with frequent fluctuations in oxygenation: a pilot clinical trial. J Pediatr. (2009) 155:640–5. 10.1016/j.jpeds.2009.04.057 19595375

[61] Hallenberger A Poets CF Horn W Seyfang A Urschitz MS. Closed-loop automatic oxygen control (CLAC) in preterm infants: a randomized controlled trial. Pediatrics. (2014) 133:e379–85. 10.1542/peds.2013-1834 24470641

[62] Johannigman JA Branson R Lecroy D Beck G. Autonomous control of inspired oxygen concentration during mechanical ventilation of the critically injured trauma patient. J Trauma. (2009) 66:386–92. 10.1097/TA.0b013e318197a4bb 19204511

[63] Arnal J-M Wysocki M Novotni D Demory D Lopez R Donati S. Safety and efficacy of a fully closed-loop control ventilation (IntelliVent-ASV(R)) in sedated ICU patients with acute respiratory failure: a prospective randomized crossover study. Inten Care Med. (2012) 38:781–7. 10.1007/s00134-012-2548-6 22460854

[64] Lellouche F Bouchard P-A Simard S L'Her E Wysocki M. Evaluation of fully automated ventilation: a randomized controlled study in post-cardiac surgery patients. Intens Care Med. (2013) 39:463–71. 10.1007/s00134-012-2799-2 23338569

[65] Johannigman JA Branson RD Edwards MG. Closed loop control of inspired oxygen concentration in trauma patients. J Am Coll Surg. (2009) 208:763–8. 10.1016/j.jamcollsurg.2009.01.033 19476833

[66] Saihi K Richard J-CM Gonin X Krüger T Dojat M Brochard L. Feasibility and reliability of an automated controller of inspired oxygen concentration during mechanical ventilation. Crit Care. (2014) 18:R35-R. 10.1186/cc13734 24552490 PMC4031979

[67] De Bie AJ Neto AS van Meenen DM Bouwman AR Roos AN Lameijer JR. Fully automated postoperative ventilation in cardiac surgery patients: a randomised clinical trial. Br J Anaesth. (2020) 125:739–49. 10.1016/j.bja.2020.06.037 32739044

[68] van Zanten HA Pauws SC Beks EC Stenson BJ Lopriore E Te Pas AB. Improving manual oxygen titration in preterm infants by training and guideline implementation. Eur J Pediatr. (2017) 176:99–107. 10.1007/s00431-016-2811-x 27888413 PMC5219007

[69] Armbruster J Schmidt B Poets CF Bassler D. Nurses' compliance with alarm limits for pulse oximetry: qualitative study. J Perinatol. (2010) 30:531–4. 10.1038/jp.2009.189 20010614

[70] Mao C Wong DT Slutsky AS Kavanagh BP A. quantitative assessment of how Canadian intensivists believe they utilize oxygen in the intensive care unit. Crit Care Med. (1999) 27:2806–11. 10.1097/00003246-199912000-00033 10628630

[71] Grim CCA Cornet AD Kroner A Meiners AJ Brouwers AJBW Reidinga AC. Attitudes of Dutch intensive care unit clinicians towards oxygen therapy. Neth J Med. (2020) 78:167–74. 32641541

[72] Roué JM Delpeut J d‘Hennezel A Tierrie T Barzic A L'Her E. Automatic oxygen flow titration in spontaneously breathing children: An open-label randomized controlled pilot study. Pediatr Pulmonol. (2020) 55:3180–8. 10.1002/ppul.25035 32827344

[73] Aboab J Louis B Jonson B Brochard L. Relation between PaO2/FIO2 ratio and FIO2: a mathematical description. Intensive Care Med. (2006) 32:1494–7. 10.1007/s00134-006-0337-9 16896846

[74] Tarnow-Mordi WO Hau C Warden A Shearer AJ. Hospital mortality in relation to staff workload: a 4-year study in an adult intensive-care unit. Lancet. (2000) 356:185–9. 10.1016/S0140-6736(00)02478-8 10963195

[75] Darbyshire JL Young JD. An investigation of sound levels on intensive care units with reference to the WHO guidelines. Crit Care. (2013) 17:R187. 10.1186/cc12870 24005004 PMC4056361

[76] Smith HAB Besunder JB Betters KA Johnson PN Srinivasan V Stormorken A. 2022 society of critical care medicine clinical practice guidelines on prevention and management of pain, agitation, neuromuscular blockade, and delirium in critically ill pediatric patients with consideration of the ICU environment and early mobility. Pediatr Crit Care Med. (2022) 23:e74–e110. 10.1097/PCC.0000000000002873 35119438

[77] Martin D Johns C Sorrell L Healy E Phull M Olusanya S. Effect of skin tone on the accuracy of the estimation of arterial oxygen saturation by pulse oximetry: a systematic review. Br J Anaesth. (2024) 132:945–56. 10.1016/j.bja.2024.01.023 38368234 PMC11103098

[78] Peters MJ Gould DW Ray S Thomas K Chang I Orzol M. Conservative versus liberal oxygenation targets in critically ill children (Oxy-PICU): a UK multicentre, open, parallel-group, randomised clinical trial. Lancet. (2024) 403:355–64. 10.2139/ssrn.4519546 38048787

