# Supplementary figures and images for: Closed–loop oxygen usage during invasive mechanical ventilation of pediatric patients (CLOUDIMPP): a randomized controlled cross-over study
Source: Front Med (Lausanne). 2024 Sep 10;11:1426969. doi: 10.3389/fmed.2024.1426969 (PMC11420134; doi:10.3389/fmed.2024.1426969)

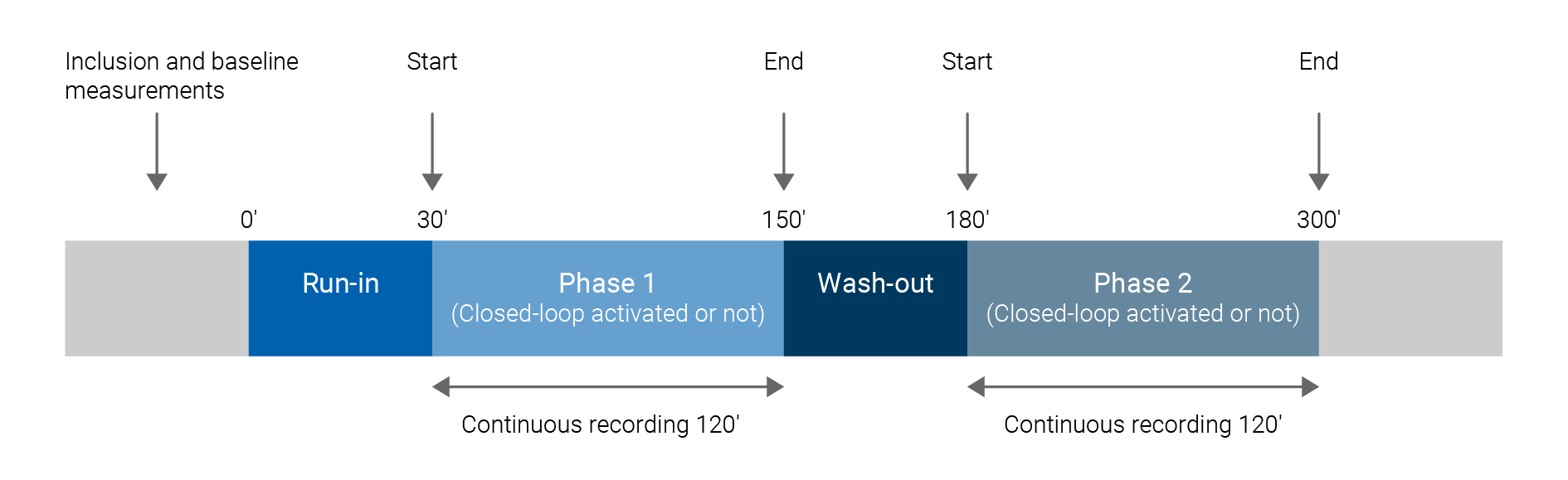

Supplement: Supplementary Figure 1 — Trial flow diagram. [file Image_1.PNG]
