# Supplementary material for: Closed–loop oxygen usage during invasive mechanical ventilation of pediatric patients (CLOUDIMPP): a randomized controlled cross-over study
Source: Front Med (Lausanne). 2024 Sep 10;11:1426969. doi: 10.3389/fmed.2024.1426969 (PMC11420134; doi:10.3389/fmed.2024.1426969)
Supplement: Supplementary Table 1 — SpO2 predefined targets. [file Data_Sheet_1.PDF]

Supplement Table 1, SpO2 (Peripheral oxygen saturation) predefined target ranges

Group	Unacceptably low	Suboptimally low	Optimal	Suboptimally high	Unacceptably high
**Highest Clinical Target**	< 91%	≥ 91% and < 94%	≥ 94% and ≤ 98%; ≥ 94% if FiO2 = 0.21	> 98% and ≤ 99	> 99%
**Higher Clinical Target**	< 90%	≥ 90% and < 93	≥ 93% and ≤ 97%; ≥ 93% if FiO2 = 0.21	> 97% and ≤ 99	> 99%
**Lower Clinical Target**	< 89%	≥ 89% and < 92	≥ 92% and ≤ 96%; ≥ 92% if FiO2 = 0.21	> 96% and ≤ 98	> 98%
**Lowest Clinical Target**	< 85%	≥ 85% and < 88%	≥ 88% and ≤ 92%; ≥ 88% if FiO2 = 0.21	> 92% and ≤ 95	> 95%

FiO2: Fraction of inspired oxygen
